# The Eigenvalue Complexity of Sequences in the Real Domain

**DOI:** 10.3390/e21121194

**Published:** 2019-12-05

**Authors:** Lingfeng Liu, Hongyue Xiang, Renzhi Li, Hanping Hu

**Affiliations:** 1 School of Software, Nanchang University, Nanchang 330031, China; xhyforwhat@163.com (H.X.); lerezz@163.com (R.L.); 2 School of Automation, Huazhong University of Science & Technique, Wuhan 430074, China; hphu@hust.edu.cn

**Keywords:** eigenvalue, real number sequences, complexity

## Abstract

The eigenvalue is one of the important cryptographic complexity measures for sequences. However, the eigenvalue can only evaluate sequences with finite symbols—it is not applicable for real number sequences. Recently, chaos-based cryptography has received widespread attention for its perfect dynamical characteristics. However, dynamical complexity does not completely equate to cryptographic complexity. The security of the chaos-based cryptographic algorithm is not fully guaranteed unless it can be proven or measured by cryptographic standards. Therefore, in this paper, we extended the eigenvalue complexity measure from the finite field to the real number field to make it applicable for the complexity measurement of real number sequences. The probability distribution, expectation, and variance of the eigenvalue of real number sequences are discussed both theoretically and experimentally. With the extension of eigenvalue, we can evaluate the cryptographic complexity of real number sequences, which have a great advantage for cryptographic usage, especially for chaos-based cryptography.

## 1. Introduction

Sequence complexity can be regarded as a series of measures that depicts the different characteristics of sequences. For cryptographic uses, the most important complexity measures of sequences are linear complexity, Lempel–Ziv (LZ) complexity, eigenvalue, and nonlinear complexity. The nonlinear complexity of a sequence *y* is an important measure, and it is defined as the length of the shortest Feedback Shift Register (FSR) that generates *y*. For the shortest Linear Feedback Shift Register (LFSR), it is referred to as the linear complexity. These two measures have been studied for many decades [1,2,3,4,5,6]. In addition, Lempel and Ziv proposed another well known complexity measure for a given sequence, which is called the LZ complexity [7]. The complexity is related to the number of distinct phrases and the rate of their occurrence along the sequence. In the same study, the eigenvalue was provided from a similar aspect as well, while the eigenvalue profile more closely reflected the rate of vocabulary growth than the LZ complexity. The relationship between LZ complexity and nonlinear complexity was studied in [8], which shows that these two complexity measures are converse in a sense.

For all these complexities, there exists a premise, which is that the measured sequences should be on the finite field. This implies that the cardinality of the state set of the sequences should be finite. For linear complexity and nonlinear complexity, the cardinality of the state set is always set to be two, which corresponds to a binary sequence. The Lempel–Ziv complexity and the eigenvalue can measure the sequences with *N* symbols, where *N* is finite. [9] studied the relationship between the eigenvalue and Shannon’s entropy of finite symbol sequences. The authors of [10] studied the relationship between the nonlinear complexity and Shannon’s entropy of random binary sequences. Moreover, the authors of [11] investigated a method to construct finite length sequences with the large nonlinear complexity on the finite field. In addition, the authors of [12] used the Lempel–Ziv complexity as a nonlinear analysis tool on the characterization of the effects of sleep deprivation on the electroencephalogram, etc. To the best of our knowledge, most of the studies in these complexity measures, including theoretical analyses and practical applications, were subjected to this constraint.

Nowadays, many physical systems can be used in cryptography for its complex dynamical properties, such as chaos-based cryptography [13,14,15,16,17,18,19,20]. However, chaotic systems are based on the real number field **R***^n^*, and the cardinality of the state variable is infinite. Currently, we always prove a chaos-based secure cryptographic algorithm based on its high dynamical complexity. However, the dynamical complexity is not completely equal to the cryptographic complexity. Thus, the security of chaos-based cryptographic algorithms is not guaranteed by cryptography researchers [21]. 

In order to overcome this weakness of chaos-based cryptography, we should evaluate the complexity of chaotic sequences in a cryptographic way. However, all the cryptographic complexity measures currently used are only available for finite symbol sequences. Thus, we should extend the cryptographic complexity from the finite field to the real number field. In this paper, we mainly focused on the eigenvalue complexity, extending this measure from the finite field to the real number field to evaluate the cryptographic properties of real number sequences. The probability distribution, expectation, and variance of the eigenvalue of real number sequences are discussed both theoretically and experimentally.

The rest of this paper is organized as follows. In Section 2, a brief introduction for the eigenvalue is presented and its extension to the real number field is proposed. The eigenvalue of two kinds of real number sequences are discussed in Section 3, including the uniformly distributed random sequence and the logistic chaotic sequence. In Section 4, four kinds of chaotic sequences are evaluated and compared by using the extended eigenvalue measure. Finally, Section 5 concludes the whole paper.

## 2. Eigenvalue for Real Number Sequences

### 2.1. Eigenvalue for Binary Sequences

The eigenvalue was first proposed by Lempel and Ziv in [7], which described the number of words occurring from a particular parsing procedure of the sequence. Here, we simply summarized the definition of eigenvalue. 

Let **F**_2_ denote the binary field and *x^N^* = *x*_0_*x*_1_*x*_2_…*x_N_*_−1_ be s binary sequence with length *N*. *x_i_^j^* is denoted as the tuple *x_i_*…*x_j_* in the sequence, *i* ≤ *j*. The prefix and suffix of sequence *x^N^* is defined as *x*_0_*^j^* and *x_j_^N^^−1^*, respectively. When *j* < *N*-1, they refer to proper prefix and proper suffix, respectively. The vocabulary of a sequence *x^N^* is the set consisting of all tuples. If a tuple *x_i_^j^* does not belong to the vocabulary of a proper prefix of *x^N^*, it is called an eigenword. The eigenvalue of a sequence equals the total number of eigenwords. The eigenvalue profile of *x^N^* is the integer-valued sequence determined by *k*(*y^i^*), *i* = 1, 2,…, *N*.

**Proposition** **1** **([7]).** *The eigenvalue* *k(y^N^)* *of sequence* *y^N^* *equals the least* *l* *such that* *y^N^* *is reproducible by* *y^l^.*

**Example** **1.** *Consider a binary sequence x^6^ = 010110. The vocabulary of this sequence is {0, 1, 01, 10, 11, 010, 101, 011, 110, 0101, 1011, 0110, 01011, 10110, 010110}. The eigenwords set is {110, 0110, 10110, 010110}. Hence, the eigenvalue of x^6^ is 4, and the eigenvalue profile of x^6^ is 122244.*

Based on the definition of the eigenvalue that we have, the eigenvalue of *x^N^* equals to *k* if, and only if, the following two conditions hold.

(1) The tuple *x_k_*_−1_*x_k_*…*x_N_*_−1_ does not belong to the vocabulary of a proper prefix of *y^N^*.
(2) The tuple *x_k_x_k_*_+1_…*x_N_*_−1_ belongs to the vocabulary of a proper prefix of *y^N^*.

Obviously, the definition of the eigenvalue is available for binary sequences, and can be extended to finite symbol sequences at most. However, the chaotic signals are defined on the real number domain, which cannot be measured by this index. Therefore, extending the eigenvalue measure to the real number domain is beneficial to chaos-based cryptography and many other aspects as well. 

### 2.2. Eigenvalue of Sequences in the Real Domain

As we know, the eigenvalue measures the rate of growth of its vocabulary of a sequence. However, strictly speaking, for a real random or chaotic sequence, there will not exist a tuple that occurs more than once. Thus, we cannot judge a tuple based on whether it belongs or does not belong to the vocabulary of a proper prefix.

In the real number field, the Euclidean distance is always used to judge whether two points are close or not. Furthermore, in the dynamics analysis, many measures are based on the Euclidean distance, such as the Lyapunov exponent, Kolmogorov entropy, and embedding dimension. Therefore, in this paper, we used the Euclidean distance *d* to judge whether the new generated signal was repeated or not. Assume that the sequence *y^N^* = *y*_0_*y*_1_*y*_2_…*y_N_*_−1_ is a sequence in the real domain, *y_i_*∈**R**. The state *y_j_* is regarded to be identical with *y_i_* if |*y_j_* − *y_i_*| < *d*, where *i* < *j*, *x_i_* is a state in the prefix *y*_0_*^j^*. *d* is defined as the undifferentiated distance that is used to judge whether two real numbers can be regarded as the same. On this basis, we can judge whether the tuple in a sequence belongs to the vocabulary of a proper prefix or not, and whether the eigenvalue can be used in the real number sequences.

Consider a random real number sequence *x^N^* = *x*_0_*x*_1_*x*_2_…*x_N_*_−1_, where x_i_∈(*a*, *b*). Assume that the distribution function of this sequence is *p*(*x*). The probability *P*(*d*) of the distance of two states *x_i_* and *x_j_*, being larger than *d*, can be calculated as

(1) $$\begin{array}{c} P(d)=P(\left|x-y\right|>d)=\int_{a}^{a+d}p(x)\int_{x+d}^{b}p(y)dydx+\int_{b-d}^{b}p(x)\int_{a}^{x-d}p(y)dydx\\ +\int_{a+d}^{b-d}p(x)\left(\int_{a}^{x-d}p(y)dy+\int_{x+d}^{b}p(y)dy\right)dx \end{array}$$

As shown in Equation (1), the probability *P*(*d*) can be calculated as the sum of three probabilities. One is the probability of *x_i_*∈(*a*, *a+d*) and *x_j_*∈(*x_i_*+*d*, *b*); one is the probability of *x_i_*∈(*b*-*d*, *b*) and *x_j_*∈(*a*, *x_j_*-*d*); and one is the probability of *x_i_*∈(*a*+*d*, *b-d*) and *x_j_*∈(*a*, *x_i_-d*) or (*x_i_+d*, *b*). Obviously, the probability *P*(*d*) is influenced by the undifferentiated distance *d*. The states *x_i_* and *x_j_* in the sequence are regarded to be identical with the probability 1-*P*(*d*). Thus, the probability of tuple *x_i_x_i_*_+1_…*x_N_*_−1_ belongs to the vocabulary *M* of its proper prefix, which can be written as

(2) $$P\left(x_{i}x_{i+1}\ldots x_{N-1}\in M\right)=1-{\left(1-{\left(1-P\left(d\right)\right)}^{N-i}\right)}^{i}$$

According to conditions (1) and (2), the probability of *k*(*x^N^*) = *k* can be written as

(3) $$\begin{array}{ll} P\left(k(y^{N})=k\right) & =P\left(x_{k}x_{k+1}\ldots x_{N-1}\in M,x_{k-1}x_{k}\ldots x_{N-1}\notin M\right)\\ & =1-P\left(x_{k}x_{k+1}\ldots x_{N-1}\in M,x_{k-1}x_{k}\ldots x_{N-1}\in M\right)\\ & \;-P\left(x_{k}x_{k+1}\ldots x_{N-1}\notin M,x_{k-1}x_{k}\ldots x_{N-1}\in M\right)\\ & \;-P\left(x_{k}x_{k+1}\ldots x_{N-1}\notin M,x_{k-1}x_{k}\ldots x_{N-1}\notin M\right) \end{array}$$

As we know, if the tuple *x_k_*_−1_*x_k_*…*x_N_*_−1_∈*M*, the tuple *x_k_x_k_*_+1_…*x_N_*_−1_ must belong to *M* as well. Thus, we have

(4) $$P\left(x_{k}x_{k+1}\ldots x_{N-1}\in M,x_{k-1}x_{k}\ldots x_{N-1}\in M\right)=P\left(x_{k-1}x_{k}\ldots x_{N-1}\in M\right)$$

(5) $$P\left(x_{k}x_{k+1}\ldots x_{N-1}\notin M,x_{k-1}x_{k}\ldots x_{N-1}\in M\right)=0$$

Furthermore, once the tuple *x_k_x_k_*_+1_…*x_N_*_−1_ does not belong to *M*, the tuple *x_k_*_−1_*x_k_*…*x_N_*_−1_ will not belong to *M* either. Thus, we have

(6) $$P\left(x_{k}x_{k+1}\ldots x_{N-1}\notin M,x_{k-1}x_{k}\ldots x_{N-1}\notin M\right)=P\left(x_{k}x_{k+1}\ldots x_{N-1}\notin M\right)$$

According to Equations (4)–(6), Equation (3) can be simplified as

(7) $$\begin{array}{ll} P\left(k(y^{N})=k\right) & =1-P\left(x_{k-1}x_{k}\ldots x_{N-1}\in M\right)-0-P\left(x_{k}x_{k+1}\ldots x_{N-1}\notin M\right)\\ & =P\left(x_{k}x_{k+1}\ldots x_{N-1}\in M\right)-P\left(x_{k-1}x_{k}\ldots x_{N-1}\in M\right) \end{array}$$

When Equation (2) is put into Equation (7), the probability of *k*(*x^N^*) = *k* can be written as

(8) $$P\left(k(y^{N})=k,d\right)={\left(1-{\left(1-P\left(d\right)\right)}^{N-k+1}\right)}^{k-1}-{\left(1-{\left(1-P\left(d\right)\right)}^{N-k}\right)}^{k}$$

Based on Equation (8), the expectation and variance of the eigenvalue of the real number random sequence *x^N^* can be written as

(9) $$\begin{array}{ll} E\left(k(x^{N}),d\right)=\sum_{k=1}^{N}k\cdot P\left(k(x^{N})=k\right)= & \sum_{k=1}^{N}k\left({\left(1-{\left(1-P\left(d\right)\right)}^{N-k+1}\right)}^{k-1}-{\left(1-{\left(1-P\left(d\right)\right)}^{N-k}\right)}^{k}\right)\\ & =1+\sum_{k=1}^{N-1}{\left(1-{\left(1-P(d)\right)}^{N-k}\right)}^{k} \end{array}$$

and

(10) $$D\left(k\left(x^{N}\right),d\right)=\sum_{k=1}^{N}{\left(k-E\left(k\left(x^{N}\right),d\right)\right)}^{2}\cdot P\left(k(x^{N})=k,d\right)$$

respectively.

Next, we use the extended eigenvalue to measure the complexity of uniformly distributed random sequences and logistic chaotic sequences.

## 3. Two Examples

### 3.1. Eigenvalue of Uniformly Distributed Random Sequence

Consider a uniformly distributed random sequence, whose distributed function is

(11) $$f\left(x\right)=\frac{1}{b-a},\;a<x<b$$

Without loss of generality, we can limit the region from (*a*, *b*) into (0, 1). The corresponding distributed function is *f*(*x*) = 1, 0 < *x* < 1. When the distribution function is brought into Equation (1), we have

(12) $$P(d)=P(\left|x-y\right|>d)=d^{2}-2d+1$$

Therefore, the probability of *k*(*x^N^*) = *k* can be depicted as

(13) $$P\left(k(y^{N})=k,d\right)={\left(1-{\left(2d-d^{2}\right)}^{N-k+1}\right)}^{k-1}-{\left(1-{\left(2d-d^{2}\right)}^{N-k}\right)}^{k}$$

**Figure 1:**
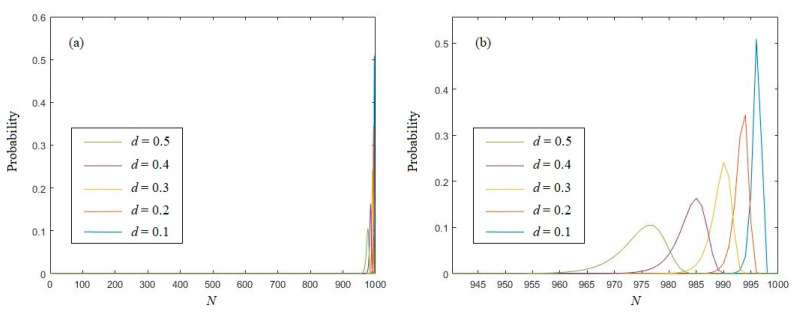
(**a**) The probability distribution of the eigenvalue of random real number sequences; (**b**) the enlargement of (**a**).

In order to have a more intuitive understanding, the probability distribution of the eigenvalue is depicted in Figure 1 with different undifferentiated distances. The length *N* is set to be 1000.

In Figure 1, we can see that most of the sequences’ eigenvalue are located in a relatively narrow interval. Obviously, with different distances *d*, the probabilities of *k*(*x^N^*) = *k* are different. The peak will move left with the growth of *d*, and the peak value will be gradually decreased. Thus, to evaluate the eigenvalue of a real number sequence, the choice of distance *d* is crucial. Consider that [9] has studied the eigenvalue probability of *n*-symbols’ random sequences with uniformly distributed sequences. In order to keep consistency, we should choose 2*d* − *d*^2^ = 1/*n*, and then the distance *d* should be chosen by

(14) $$d=1-\sqrt{1-1/n}$$

Therefore, we can compare with the eigenvalue of binary random sequence by choosing *d* = 0.2929, and we can compare with the eigenvalue of 3-symbols random sequence by choosing *d* = 0.1835, and we can compare with the eigenvalue of 4-symbols random sequence by choosing *d* = 0.1340, etc. 

**Figure 2:**
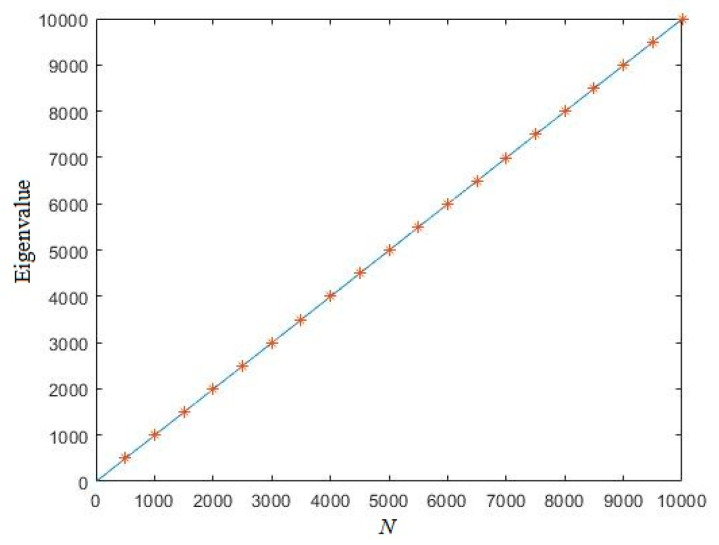
The Eigenvalue of random sequences (the solid line denotes the theoretical curve of the expectation of the eigenvalue for random real number sequences; symbol ‘*’ denotes the eigenvalue of the randomly generated sequence).

Based on the distribution of eigenvalues, the expectation of the eigenvalue for random real number sequences can be approximately calculated as

(15) $$\begin{array}{ll} E\left(k(x^{N}),d\right) & =\sum_{k=1}^{N}k\cdot P\left(k(x^{N})=k,d\right)\\ & =\sum_{k=1}^{N}k\left({\left(1-{\left(2d-d^{2}\right)}^{N-k+1}\right)}^{k-1}-{\left(1-{\left(2d-d^{2}\right)}^{N-k}\right)}^{k}\right)\\ & =1+\sum_{k=1}^{N-1}{\left(1-{\left(2d-d^{2}\right)}^{N-k}\right)}^{k}\\ & \approx N{-\log}_{1/(2d-d^{2})}N \end{array}$$

for moderate–large *N*. Set *N* = 10,000, a uniformly distributed random sequence is randomly generated. Figure 2 shows the eigenvalue of this sequence. In Figure 2, we can see that all the numerical results are near the theoretical curve we derived in Equation (15), which indicates that the expectation of eigenvalue of random real number sequences is correct.

Based on Equation (10), the variance of the eigenvalue of random real number sequences can be approximately written as

(16) $$D\left(k\left(x^{N}\right),d\right)=\sum_{k=1}^{N}{\left(k-N+\log_{1/(2d-d^{2})}N\right)}^{2}\cdot P\left(k(x^{N})=k,d\right)$$

**Figure 3:**
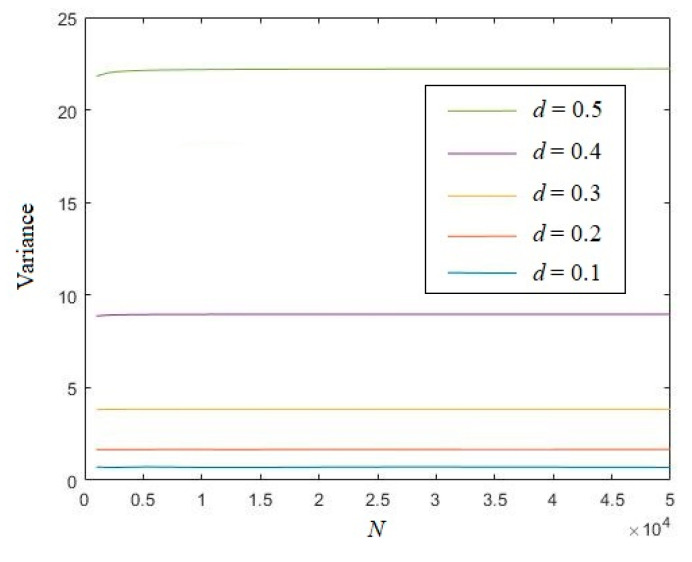
Variances of the eigenvalue of a random real number sequence.

Set length *N* from 1000 to 50,000. Figure 3 shows that for different distances *d*, the variances of the eigenvalue are all quite stable with the growth of length *N*.

### 3.2. Eigenvalue of Logistic Chaotic Sequence

Consider the following logistic chaotic map,

(17) $$y_{i+1}=1-2y_{i}^{2}$$

where *y_i_*∈(−1, 1) is the state variable. For an initial condition *y*_0_, we can generate a chaotic sequence *y*_0_*y*_1_…*y_n s_* according to the iteration. The distribution function of Equation (17) is [22]

(18) $$f(y)=\frac{1}{\pi \sqrt{1-y^{2}}},\;-1\leq y\leq 1$$

According to Equation (1), the probability *P*(*d*) of the distance of two states, *y_i_* and *y_j_*, is larger than d and can be calculated as

(19) $$\begin{array}{ll} P(d)=\int_{-1}^{-1+d}f(y_{i}) & \int_{x+d}^{1}f(y_{j})dy_{j}dy_{i}+\int_{1-d}^{1}f(y_{i})\int_{-1}^{x-d}f(y_{j})dy_{j}dy_{i}\\ & +\int_{-1+d}^{1-d}f(y_{i})\left(\int_{-1}^{x-d}f(y_{j})dy_{j}+\int_{x+d}^{1}f(y_{j})dy_{j}\right)dy_{i}\\ & =\frac{1}{4}+\frac{\arcsin(1-d)}{\pi }+\frac{\arcsin^{2}(1-d)}{\pi^{2}} \end{array}$$

**Figure 4:**
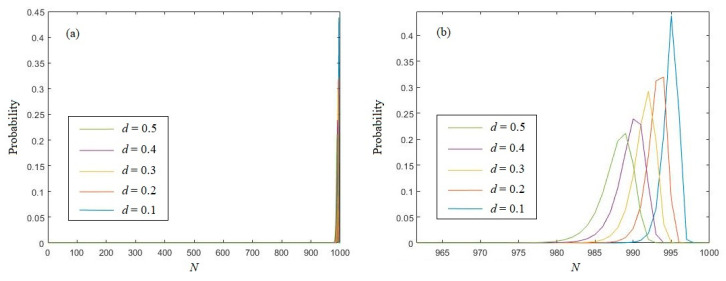
(**a**) The probability distribution of the eigenvalue of logistic chaotic sequences; (**b**) the enlargement of (**a**).

When Equation (19) is brought into Equation (8), the probability of *k*(*x^N^*) = *k* for the logistic chaotic sequence can be easily calculated. Figure 4 depicts the probability distribution of the eigenvalue of logistic chaotic sequences with different *d* values. In Figure 4, we can see that, as with random sequences, the eigenvalue are also located in a relatively narrow interval, and the peak will move left with the growth of *d*. The peak value will gradually be decreased as well.

**Figure 5:**
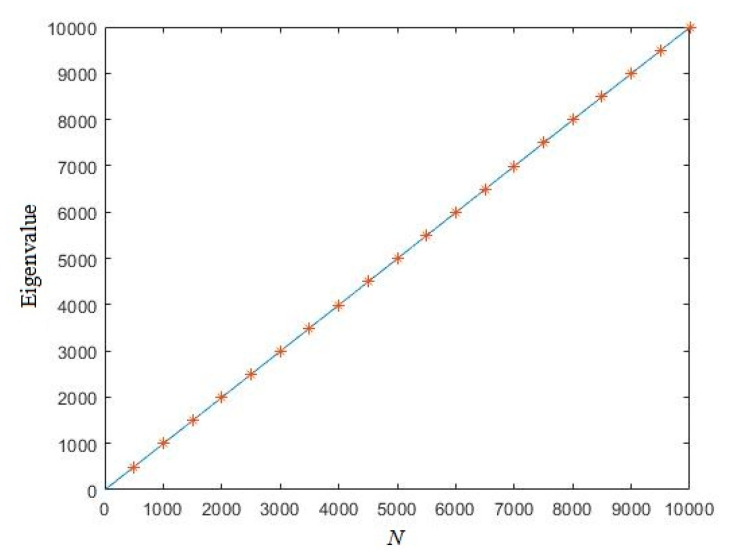
The eigenvalue of the logistic sequence (the solid line denotes the theoretical curve of expectation of eigenvalue for random real number sequences; symbol ‘*’ denotes the eigenvalue of the generated logistic sequence).

Based on Equation (9), the expectation of the eigenvalue of the logistic chaotic sequence can be written as

(20) $$\begin{array}{ll} E\left(k(x^{N}),d\right) & =\sum_{k=1}^{N}k\cdot P\left(k(x^{N})=k,d\right)\\ & =\sum_{k=1}^{N}k\left(\begin{array}{l} {\left(1-{\left(\frac{3}{4}-\frac{\arcsin(1-d)}{\pi }-\frac{\arcsin^{2}(1-d)}{\pi^{2}}\right)}^{N-k+1}\right)}^{k-1}\\ -{\left(1-{\left(\frac{3}{4}-\frac{\arcsin(1-d)}{\pi }-\frac{\arcsin^{2}(1-d)}{\pi^{2}}\right)}^{N-k}\right)}^{k} \end{array}\right)\\ & =1+\sum_{k=1}^{N-1}{\left(1-{\left(\frac{3}{4}-\frac{\arcsin(1-d)}{\pi }-\frac{\arcsin^{2}(1-d)}{\pi^{2}}\right)}^{N-k}\right)}^{k}\\ & \approx N{-\log}_{4\pi^{2}/(3\pi^{2}-4\pi \arcsin(1-d)-4\arcsin^{2}(1-d))}N \end{array}$$

for moderate–large *N*. When we randomly select an initial condition, Figure 5 shows the eigenvalue of this generated logistic sequence. Obviously, the eigenvalues of this sequence are all around the theoretical curve we derived in Equation (20).

**Figure 6:**
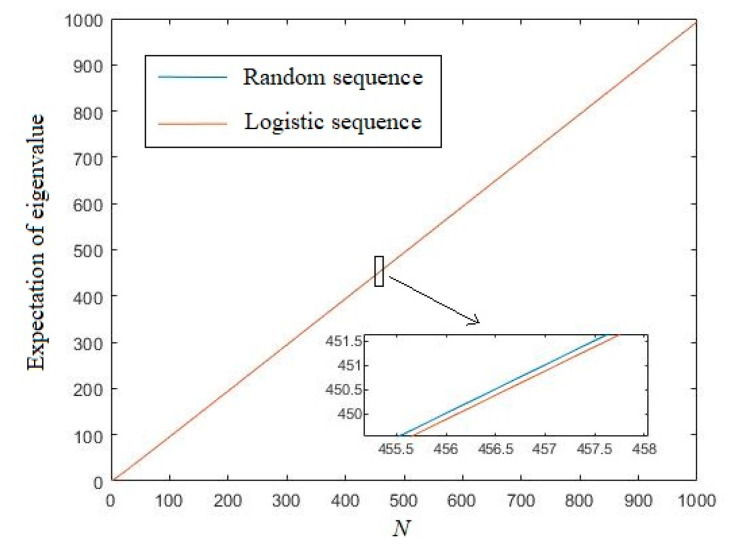
The expectation of the eigenvalue of logistic sequences and random sequences.

Figure 6 depicts the comparison of the expectation of the eigenvalue of logistic sequences and uniformly distributed random sequences under the same undifferentiated distance *d* = 0.1. In Figure 6, we can see that there are almost no differences among the expectation eigenvalue of the logistic sequences and random sequences. After enlarging, we can see that the eigenvalue of logistic sequences is just a little lower than the random sequence, which implies that the logistic sequence cannot be regarded as a perfect random sequence in this sense. For other undifferentiated distances, the results are similar. Therefore, we omit them here to avoid redundancy.

Correspondingly, the variance of the eigenvalue of logistic sequences can be approximately written as

(21) $$D\left(k\left(x^{N}\right),d\right)=\sum_{k=1}^{N}{\left(k-N+\log_{4\pi^{2}/(3\pi^{2}-4\pi \arcsin(1-d)-4\arcsin^{2}(1-d))}N\right)}^{2}\cdot P\left(k(x^{N})=k,d\right)$$

**Figure 7:**
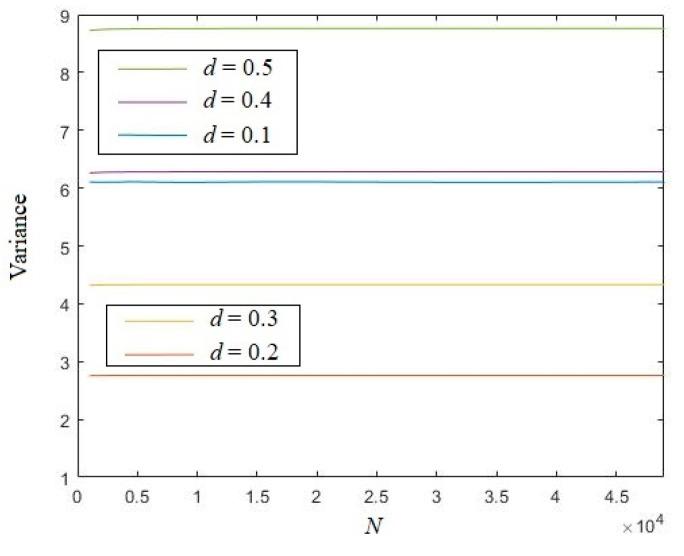
The variance of the eigenvalue of logistic sequences.

The variances of the eigenvalue of logistic sequences with different distances *d* are depicted in Figure 7. This figure indicates that for every distance, the eigenvalue of logistic sequences are all stable with the growth of length *N*. 

## 4. Measure the Complexity of Chaotic Sequences 

With the extension of eigenvalue, we can use this complexity measure to evaluate the cryptographic characteristics of different chaotic sequences. Here, the following four kinds of 1-D chaotic sequences are generated and compared.

- Chebyshev map
  Chebyshev map can be written as
  (22) $$x_{i+1}=\cos(a\cdot \arccos(x_{i}))$$
  where *x_i_*∈(−1, 1) is the state variable, *a* is the control coefficient. The Chebyshev map will be chaotic since *a*≧2. In this test, we always set *a* = 3.
- Sine map
  Sine map can be mathematically described as
  (23) $$x_{i+1}=r\sin(\pi x_{i})$$
  where *r*∈(0, 1] is the control parameter. In this test, we set *r* = 2 to make the Sine map chaotic.
- Tent map
  Tent map is a kind of piece-wise function, which can be described as
  (24) $$x_{i+1}=\left\{ \begin{array}{ll} x_{i}/p, & x_{i}\in [0,p)\;\\ (1-x_{i})/(1-p), & x_{i}\in [p,1] \end{array}\right.$$
  where *p*∈(0, 1) is the control parameter. Particularly, when *p* = 0.5, the generated sequence will quickly fall into a short cycle. Therefore, we always set *p* = 0.49 in this test.
- Logistic map
  The Logistic map has already been described in Equation (17), which we omitted here to avoid redundancy.

**Figure 8:**
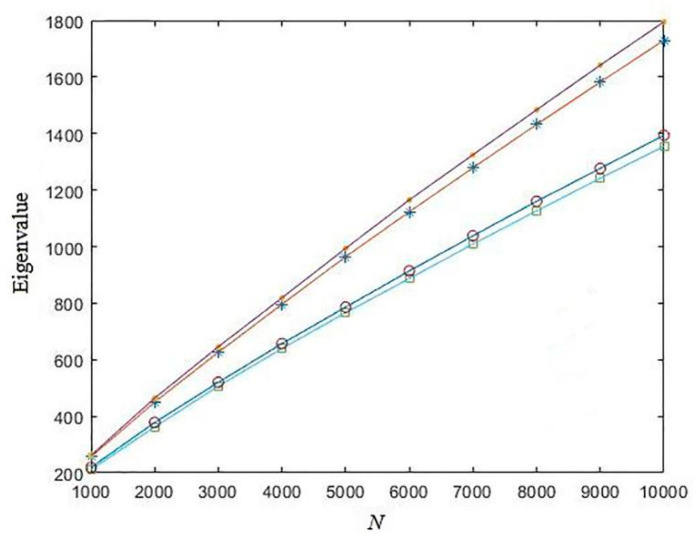
The eigenvalue of different chaotic sequences. (The asterisk represents the eigenvalue of the Chebyshev chaotic sequence; the solid point represents the eigenvalue of the sine chaotic sequence; the square represents the eigenvalue of the Tent chaotic sequence; the hollow point represents the eigenvalue of the logistic chaotic sequence).

Since the state variables of these four maps are in different domains, for consistency, we first compressed them to the identical interval (0, 1). When *d* = 0.1, the eigenvalue of these four kinds of chaotic sequences are depicted in Figure 8. Figure 8 shows that the chaotic sequences generated by the sine map have the largest eigenvalue, whereas the chaotic sequences generated by the Tent map have the lowest eigenvalue. For other distances *d*, the results are similar. 

Thus, it can be seen that with the extended eigenvalue, we can evaluate the cryptographic complexity of real number sequences effectively. However, it should be noted that this result does not imply that the Sine map is better than other chaotic maps in cryptographic application. On the one hand, the eigenvalue is only one of the cryptographic complexity measures; on the other hand, the eigenvalue value is influenced by the control parameter of chaotic maps. For example, the eigenvalue of the Sine chaotic sequence will be lower than the eigenvalue of the Chebyshev chaotic sequence when *r* = 1.

## 5. Conclusions

In order to evaluate the cryptographic complexity of real number sequences, in this paper, we extended the so-called eigenvalue from the binary field to the real number field. The extended eigenvalue was influenced by the undifferentiated distance, and we gave an exact value of this distance corresponding to the *N*-symbol sequences. Both uniformly distributed random sequences and logistic sequences were used as examples. The probability distribution, expectation and variance of these two kinds of real number sequences were discussed both theoretically and experimentally. With the extension of eigenvalue, we could evaluate the cryptographic complexity of real number sequences, which has a great advantage for cryptographic usage, especially for chaos-based cryptography. Furthermore, four kinds of chaotic sequences were evaluated by this extended complexity measure, which indicates that our study is effective and of great interest.
